# Aktuelle Entwicklungen der nanopartikelbasierten Tumortherapie bei Kopf-Hals-Tumoren

**DOI:** 10.1007/s00106-025-01633-0

**Published:** 2025-05-19

**Authors:** Stefan Hansen, Benjamin Kansy, Sven Brandau, Matthias Epple, Stephan Lang

**Affiliations:** 1 Hals-Nasen-Ohrenpraxis am Waldweg, Waldweg 1, 37073 Göttingen, Deutschland; 2 Klinik für Hals-Nasen-Ohrenheilkunde, Universitätsklinikum Essen, Universität Duisburg-Essen, Duisburg-Essen, Deutschland; 3 Institut für Anorganische Chemie, Universität Duisburg-Essen, Duisburg-Essen, Deutschland

**Keywords:** Nab-Paclitaxel, Calciumphosphat-Nanopartikel, Theranostik, Nanobiomedizin, Zielgerichtete Krebstherapie, SPION, Calcium phosphate nanoparticles, Theranostic, Nanomedicine, Targeted therapy

## Abstract

Die rasante Verbreitung und Anwendung von Nanopartikeln in der Medizin haben zu einer Vielzahl von experimentellen und klinischen Studien insbesondere in der onkologischen Forschung beigetragen. Innerhalb dieser sog. Nanomedizin haben sich unterschiedliche Schwerpunkte etabliert. Diese orientieren sich zum einen an den chemischen, physikalischen und biologischen Eigenschaften unterschiedlichster Nanopartikel und zum anderen an den möglichen spezifischen Anwendungen wie beispielsweise Medikamententransport, Strahlentherapie, In-vivo-Monitoring von Wirkstoffen im Tumor oder auch immunmodulierende Wirkungen. Nanopartikel können zudem funktionalisiert werden, indem bestimmte Faktoren wie Antikörper zur spezifischen Adressierung eines Zielgewebes oder die Kopplung von Chemotherapeutika die intrinsische antitumorale Wirkung von Nanopartikeln potenzieren. In der vorliegenden Arbeit wird ein aktueller Überblick über die Entwicklungen und Anwendungen von Nanopartikeln in der Kopf-Hals-Onkologie gegeben.

## Abstract (en)

The rapid expansion and application of nanoparticles in medicine has meanwhile contributed to a large number of experimental and clinical studies, especially in cancer research. Numerous different fields within nanomedicine have now become established more clearly. These are based on the one hand on the properties of different types of nanoparticles (chemical, physical, and biological) and on the other hand on the possible specific applications such as drug carrier, radioenhancer, in vivo monitoring of drug distribution within the tumor, or tumor-specific immune-modulating effects. Nanoparticles can also be functionalized by multiple properties potentiating their intrinsic antitumoral effects, such as coupling of antibodies to address target tissue or coupling to chemotherapeutic agents. In this work, an up-to-date overview of the developments and applications related to nanoparticles in head and neck cancer is given.

Als Nanopartikel bezeichnet man definitionsgemäß organische oder anorganische Teilchen mit einer Größe von 1 bis 100 nm. Gleichwohl gibt es Nanopartikel wie etwa polymere Nanopartikel oder Lipidnanopartikel, welche auch mehrere Hundert Nanometer Größe erreichen können (Abb. 1). In dieser Nanoskalierung haben die Teilchen besondere chemische, physikalische und biologische Eigenschaften, die sich vollkommen von denen des Ursprungsmaterials unterscheiden können. Die besonderen Eigenschaften von Nanopartikeln werden vor allem durch ihre vergleichsweise große Oberfläche im Verhältnis zu ihrem Volumen hervorgerufen. Darüber hinaus beeinflussen auch Struktur, Form, Ladungszustand und Oberflächenbeschaffenheit die Eigenschaften von Nanopartikeln [11]. Einige Beispiele von Nanopartikeln als Trägersubstanz finden sich in Abb. 2.

**Abb. 1:**
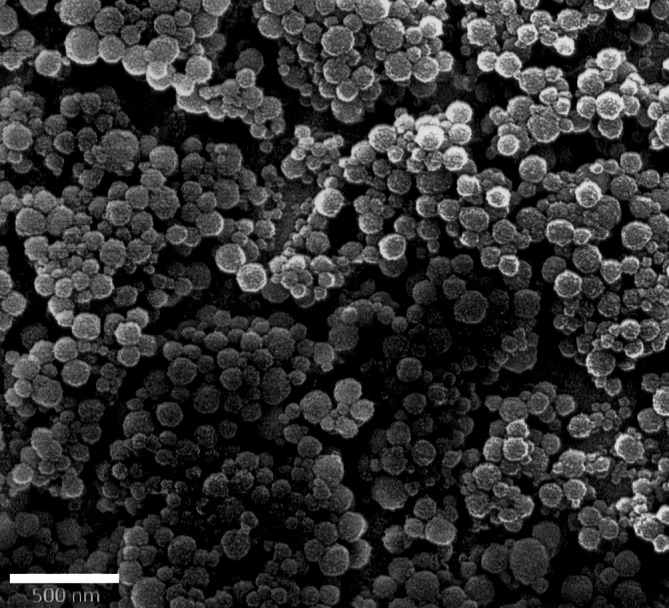
Rasterelektronenmikroskopische Aufnahme von Calciumphosphat-Nanopartikeln (*Balken* 500 nm, eigenes Bildmaterial)

**Abb. 2:**
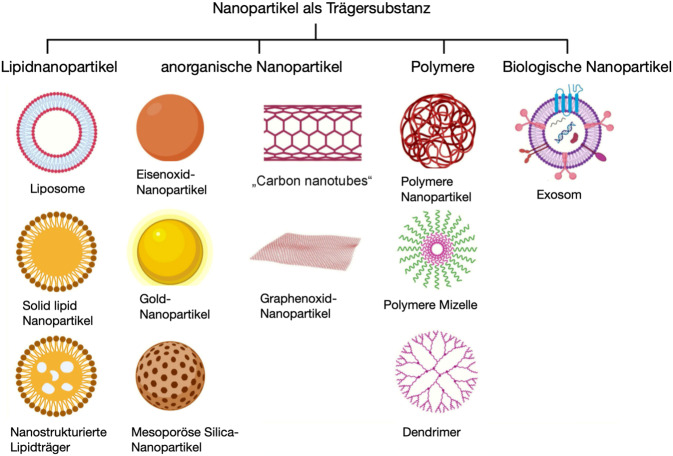
Nanopartikel, die als Trägersubstanz mögliche Anwendungen in der Tumortherapie finden. Aus [10] © 2023 by the authors. Licensee MDPI, Basel, Switzerland. (This article is an open access article distributed under the terms and conditions of the Creative Commons Attribution (*CC* BY) license [35])

Nanoeffekte kommen häufig in der Natur vor: An Fliegenbeinen befinden sich beispielsweise nanometergroße Haare, damit die Insekten an Wänden laufen können. Beim Lotuseffekt auf der Pflanzenoberfläche bewirken Nanostrukturen, dass das Wasser auf dem Blatt der Lotusblume abperlt und die Haftung von Schmutzpartikeln reduziert wird.

Als Vater der Nanotechnologie gilt Richard Feynman aufgrund seiner Visionen, die er im Jahr 1959 in dem Vortrag „There’s Plenty of Room at the Bottom“ formulierte, auch wenn der Begriff „Nanotechnologie“ im Sinne einer Werkstoffentwicklung erst 1974 von dem Ingenieurwissenschaftler Norio Taniguchi sowie die weitergehende „molekulare Nanotechnologie“ von dem Amerikaner Eric Drexler 1986 einführt worden sind [31].

Synthetisch hergestellte Nanopartikel aus Titandioxid oder Aluminiumoxid kommen heutzutage in diversen kosmetischen Produkten vor, etwa in Sonnencremes, Deos oder Zahnpasta. In Lebensmitteln wie Tomatenketchup findet sich Siliziumoxid als Verdickungsmittel. Weitere Beispiele sind Nanopartikel in Farben, Lacken und Imprägniermitteln für alle Arten von Oberflächen wie z. B. bei Kleidung.

Die Nanotechnologie drängt mit großen Schritten in klinische Anwendungen unterschiedlichster Bereiche der Medizin, die dann auch als Nanomedizin oder Nanobiotechnologie bezeichnet. Der globale Umsatz in der Nanomedizin wurde 2016 auf ca. 138,8 Mrd. US-Dollar geschätzt [32].

Wie auch in vielen anderen Fachbereichen gibt es in der HNO-Heilkunde zunehmend patientenorientierte Anwendungsstudien mit Nanopartikeln, vor allem in der Kopf-Hals-Onkologie, aber auch in der Otologie. Diese umfassen sowohl die Diagnostik in vitro und in vivo, neue Wirkstoffe durch antitumorale, nanopartikelbasierte Effekte, den Wirkstofftransport oder auch die Beschichtung von Implantaten.

## Nanopartikel als Trägersubstanz bei Kopf-Hals-Tumoren

Einige Arbeitsgruppe erwarten in der Krebstherapie zukünftig entscheidende Erfolge durch die Nanomedizin. Insbesondere nach drei Jahrzehnten der Stagnation von Überlebensraten bei Kopf-Hals-Tumoren erscheinen neue Therapieansätze mit Nanopartikeln vielversprechend, müssen jedoch ihre Wirksamkeit und Patientensicherheit erst noch abschließend unter Beweis stellen. Nichtsdestotrotz gibt es inzwischen einige klinische Studien für Kopf-Hals-Tumoren mit Anwendungen verschiedener Nanopartikel, deren Ergebnisse durchaus bemerkenswert sind.

Ein bereits in der EU und den USA für das metastasierte Mamma‑, Pankreas- und Lungenkarzinom zugelassenes Medikament mit dem Handelsnamen Abraxane beruht unter anderem auf Nanopartikeln. Es enthält das schwer lösliche Chemotherapeutikum Paclitaxel, das hierbei mit Humanalbumin-Nanopartikeln umschlossen wird. Hierdurch werden ein sonst notwendiges toxisches Lösungsmittel wie Rizinusöl und Ethanol für das Paclitaxel vermieden und zudem der Transport des Wirkstoffkomplexes über die gp60-Albuminrezeptoren in den Tumor erleichtert [14]. Eine aktuelle Übersicht von klinischen Studien zu Kopf-Hals-Karzinomen und dem Einsatz von Nanopartikeln findet sich in Tab. 1.

**Tab. 1:** Übersicht der aktuellen klinischen Studien zu Kopf-Hals-Karzinomen mit Beteiligung von Nanopartikeln [34]

Nanopartikel	Titel der Studie	Studienphase und Status	NCT-Nummer
Nanopartikel-Albumin-gebundenes Paclitaxel	Weekly Nanoparticle Albumin-Bound Paclitaxel (Abraxane) + Weekly Cetuximab + Radiation Therapy (IMRT, Intensity-Modulated Radiation Therapy) in Patients With Stage III-IVB Head and Neck Squamous Cell Carcinoma (HNSCC)	Phase-I-Studie, abgeschlossen	NCT00736619
Nanopartikel-Albumin-gebundenes Paclitaxel	Paclitaxel Albumin-Stabilized Nanoparticle Formulation and Carboplatin Followed By Chemoradiation in Treating Patients With Recurrent Head and Neck Cancer	Phase-I-Studie, abgeschlossen	NCT01847326
Nanopartikel-Albumin-gebundenes Paclitaxel	Nab-Paclitaxel, Cisplatin, and Cetuximab With Concurrent Radiation Therapy for Locally Advanced Head and Neck Cancer	Phase-I/II-Studie, abgeschlossen	NCT00851877
Nanopartikel-Albumin-gebundenes Paclitaxel	Dose-finding Study of Abraxane in Combination With Cisplatin to Treat Advanced Nasopharyngeal Carcinoma	Phase-IIa-Studie, Status unbekannt	NCT01735409
Nanopartikel-Albumin-gebundenes Paclitaxel	Induction Chemotherapy With Nab-paclitaxel, Cisplatin and Fluorouracil for Locoregionally Advanced Nasopharyngeal Carcinoma	Phase-II-Studie, Status unbekannt	NCT04004871
Nanopartikel-Albumin-gebundenes Paclitaxel	Nab-paclitaxel and Carboplatin Followed by Response-Based Local Therapy in Treating Patients With Stage III or IV HPV-Related Oropharyngeal Cancer	Phase-II-Studie, abgeschlossen	NCT02258659
Nanopartikel-Albumin-gebundenes Paclitaxel	Induction Chemotherapy With ACF Followed by Chemoradiation Therapy for Adv. Head & Neck Cancer	Phase-II-Studie, abgeschlossen	NCT01566435
Hafniumoxid-Nanopartikel	NBTXR3, Radiation Therapy, and Pembrolizumab for the Treatment of Recurrent or Metastatic Head and Neck Squamous Cell Cancer	Phase-II-Studie, Rekrutierung	NCT04862455
Hafniumoxid-Nanopartikel	JNJ-90301900 (NBTXR3) Activated by Radiotherapy With or Without Cetuximab in LA-HNSCC	Phase-III-Studie, Rekrutierung	NCT04892173
Cisplatin-inkorporierte polymere Mizelle	Study of NC-6004 in Combination With 5‑FU and Cetuximab in Patients With Head and Neck Cancer	Phase-I-Studie, beendet	NCT02817113
Paclitaxel-inkorporierte polymere Mizelle	TPC Versus GP Induction Chemotherapy for Nasopharyngeal Carcinoma	Phase-II-Studie, Rekrutierung	NCT06301165
Nanopartikel-Albumin-gebundenes Rapamycin	Nanoparticle Albumin-Bound Rapamycin in Treating Patients With Advanced Cancer With mTOR Mutations	Pilotstudie, abgeschlossen	NCT02646319

Ein in *Nature Communications* veröffentlichter Beitrag berichtet außerdem über einen interessanten Ansatz zur tumorzellspezifischen Adressierung neuer Wirkstoffe in der Krebstherapie [19]. Fucoidan ist ein Polysaccharid, welches eine hohe Affinität zu dem Zelladhäsionsmolekül P‑Selektin hat. P‑Selektin ist in Endothelzellen vieler Tumorgefäße überexprimiert und spielt zudem auch eine Rolle bei der Metastasierung. Mithilfe von Fucoidan-Nanopartikeln konnten Mizrachi et al. einen Inhibitor der Phosphoinositol-3-Kaskade namens Alpelisib in die Tumorzellen bei einem Maus-Xenograft-Kopf-Hals-Tumormodell einschleusen. Mutationen des Phosphoinositol-3-CA-Gens finden sich in mindestens 40 % HPV-positiver Kopf-Hals-Tumoren. Alpelisib wurde zuvor bereits bei Brustkrebs und soliden Tumoren untersucht und zeigte offenbar einen sehr guten antitumoralen Effekt mit jedoch relevanten Nebenwirkungen in der notwendigen Dosierung. Die Fucoidan-Nanopartikel haben nun den Wirkstoff ummantelt, an die Tumorzellen „adressiert“ und eingeschleust, sodass im Mausmodell eine hohe tumorspezifische Konzentration des Inhibitors erreicht werden konnte bei 7‑fach geringerer Dosierung und gleicher Wirksamkeit im Vergleich zur Standarddosierung von Alpelisib. Zusätzlich wurden die Nanopartikel mit einem fluoreszierenden Farbstoff gekoppelt, sodass außerdem noch ein In-vivo*-*Monitoring der Wirkstoffverteilung möglich war. Weitere Nanopartikeln, die als Trägersubstanz in onkologischen Studien verwendet werden, umfassen zudem exosomale, lipidbasierte oder polymerische Nanopartikel (Abb. 2; [10]).

Insbesondere scheinen Nanopartikel in Form von Biopolymeren günstige Eigenschaften bei der Tumoradressierung und vor allem auch der Biokompatibilität und Biodegradierung zu haben [2].

## Supraparamagnetische Eisenoxid-Nanopartikel in der Tumortherapie

Einen anderen Ansatz, um Nanopartikel als Transportvehikel zu nutzen, bieten superparamagnetische Eisenoxid-Nanopartikel (SPION). An diese können Chemotherapeutika, Radionuklide, Fluoreszenzfarbstoffe oder Antikörper allein oder in Kombination gebunden werden und über ein externes Magnetfeld im Tumor konzentriert werden. Dieses Verfahren wird auch als magnetisches „drug targeting“ bezeichnet. Hierdurch können beispielsweise bei der Diagnostik die Sensitivität einer Magnetresonanztomographie erhöht oder schwer lösliche Substanzen gebunden und im Tumor angereichert werden. In der Synthese von Eisenoxidnanopartikeln und Anwendung ist die Sektion für Experimentelle Onkologie und Nanomedizin (SEON), die an der HNO-Klinik des Uniklinikums Erlangen angesiedelt ist, führend und verfolgt hier die Translation von der Grundlagenforschung in die Klinik [1, 23, 30]. Neben der Anwendung bei Gliomen und beim Mammakarzinom scheint es zumindest beim Cisplatin-resistenten Nasopharynxkarzinom eine verbesserte Wirksamkeit zu bieten. Zusätzlich wurde in dieser Studie wurde neben der Kopplung der Nanopartikel mit Cisplatin ein endoskopisches In-vivo-Monitoring der Anreicherung durch die zusätzliche Kombination mit einem Fluoreszenzfarbstoff ermöglicht [29]. Diese Kombination aus Therapie und Diagnostik wird auch als Theranostik bezeichnet [6].

## Gold-Nanopartikel in der Tumortherapie

Eine zunehmende Verbreitung in der onkologischen Forschung zeigen insbesondere auch Gold-Nanopartikel [7]. Diese können ebenfalls als Vehikel für Wirkstoffe genutzt werden und haben je nach Form und Größe selbst intrinsische antitumoral nutzbare Eigenschaften. So akkumulieren sie beispielsweise verstärkt im Tumorgewebe aufgrund des so genannten „enhanced permeability and retention effect“ [15, 21]. Dieser entsteht vermutlich aufgrund der durchlässigeren Gefäße des Tumors infolge der verstärkten Angiogenese sowie der anschließenden Aufnahme der Nanopartikel durch perivaskuläre Zellen und Makrophagen, was dann die Clearance der Nanopartikel verzögert. Darüber hinaus haben Gold-Nanopartikel optische Eigenschaften und können mit Licht angeregt werden. Durch die Emission eines Photons entstehen hierbei Signale, die 105-fach intensiver sind als bei einem Fluoreszein-Molekül. Diese Emissionen entstehen durch kohärente Oszillation der Elektroden. Dieses als Oberflächenplasmonenresonanz bezeichnete Phänomen kann beispielsweise zur Diagnostik verwendet werden, um intraoperativ Tumorgrenzen zu identifizieren [13]. Verändert man die Form und Größe der Gold-Nanopartikel, kann statt der Photonenemission eine Absorption mit Hitzeentwicklung entstehen. Diese kann dann für eine so genannte photothermale Therapie genutzt werden [28]. Hierzu gibt es eine Phase-I-Studie, bei der Patienten mit refraktären Kopf-Hals-Tumoren oder Rezidiven eine systemische Gabe von Gold-Nanopartikeln erhalten und anschließend der Tumor lokal mit Infrarotstrahlen (nahes IF 808 nm, AuroLase^(TM)^, Nanospectra Biosciences, Inc, Houston, Texas, USA.) angeregt wird [33]. Andere Studien auf experimenteller Basis zeigen ebenfalls eine deutlich höhere lokale antitumorale Wirkung bei der Kombination von verschiedenen Gold-Nanopartikeln in Kombination mit Lasern [16]. Der Vorteil bei der Kombination von Gold-Nanopartikeln und verschiedenen Lasern ist zudem die Möglichkeit für unterschiedliche Eindringtiefen der Laser von der oberflächlichen Haut bis hin zu 10 cm tiefen Gewebeschichten. Auch andere Ablationsverfahren wie Radiofrequenz, Mikrowellen, hoch fokussierter Ultraschall und weitere photodynamische Verfahren können in Kombination mit solchen photosensitiven Nanopartikeln zumindest in vitro eine wirksame Tumorzellzerstörung erreichen [22].

In einer weiteren Eigenschaft können Gold-Nanopartikel wie auch andere Metalle die Wirkung einer applizierten Strahlendosis als so genannten Radioenhancer verstärken [4]. Im Tierversuch konnte die Strahlenempfindlichkeit durch eine Kombination von Gold-Nanopartikeln mit Cetuximab und anschließender Bestrahlung deutlich verbessert werden [25]. Theoretisch ist eine lokale Verstärkung der Strahlendosis im Tumor um 200 % möglich. Auf Basis dieser Daten konnte in einem Tumor-Mausmodell eine deutlich verbesserte Überlebensrate bei intravenöser Gabe von Nanopartikeln und anschließender Bestrahlung gezeigt werden im Vergleich zur alleinigen Bestrahlung (86 % vs. 20 % 1‑Jahres-Überlebensrate) [12]. Die Veränderung der Radiosensitivität auf zellulärer und DNA-Ebene ist keine exklusive Eigenschaft der Gold-Nanopartikel. Auch andere Nanopartikel auf Metallbasis wie Zinkoxid-Nanopartikel können in subtoxischer Dosierung zu oxidativen DNA-Schäden führen und damit die Radiosensitivität von Tumorzellen erhöhen [18].

Der bereits in klinischen Studien untersuchte Radioenhancer NBTXR3 beruht auf der chemischen Verbindung Hafniumoxid [3]. Hafnium ist ein chemisches Element mit der Ordnungszahl 72 und steht in der 4. Nebengruppe (Titangruppe) (s. auch Tab. 1).

## Nanopartikel als Strahlungsquelle

Außerdem existieren Nanopartikel, die selbst als Strahlenquelle fungieren. So wurden Radionuklide wie das Rhenium-186 entwickelt, welche in Lipid-Nanopartikel verpackt werden können, um eine bessere Penetration und Verteilung bei einer intratumoralen Applikation zu erreichen. Dieser Betastrahler mit einer Eindringtiefe von 1–4 mm und einer Halbwertszeit von 5 Tagen erreichte im Tiermodell der Ratten mit einem Xenograft-Kopf-Hals-Tumor bei intratumoraler Applikation eine lokale Intensität bis 526 Gy [9]. Zum Vergleich werden bei einer Brachytherapie bis zu 145 Gy und eine Eindringtiefe von 1–3 cm erreicht. Gleichzeitig hat Rhenium-186 auch eine geringe Gammastrahlung, sodass die Verteilung der Nanopartikel über eine Einzelphotonen-Emissions-Computertomographie (SPECT) in vivo und nichtinvasiv kontrolliert werden kann. Additiv kann noch ein Chemotherapeutikum wie Doxorubicin an den Nanopartikelkomplex gekoppelt werden [26].

## Nebenwirkungen und Immunmodulation durch die Anwendung von Nanopartikeln

**Abb. 3:**
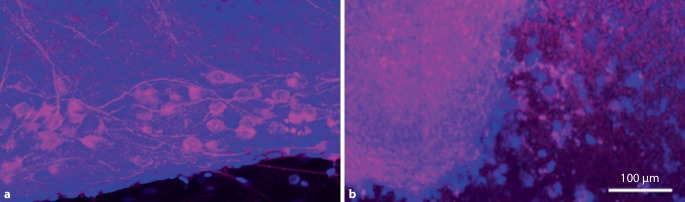
Zelltoxizität von Nanopartikeln. Immunfluoreszenzfärbung von Spiralganglienneuronen der neugeborenen Ratte sowie deren begleitenden Gliazellen auf einer Oberfläche aus Poly-Lysin allein (**a**) sowie auf Poly-Lysin mit Calciumphosphat-Nanopartikel kombiniert (Layer-by-Layer-Verfahren; **b**). Während bei Poly-Lysin normale Spiralganglienzellen erkennbar sind (*violett*), zeigen sich nahezu nur noch Zellfragmente auf der kombinierten Nanopartikel-Oberfläche (**b**). (*Violett *β‑Tubulin III, *blau *Zellkernfärbung mit 4’, 6‑Diamidino-2-Phenylindol; *Balken* 100 *µ*m, eigenes Bildmaterial)

Nanopartikel können als passives Transportmittel, als aktives Transportmittel mit spezifischer Adressierung an Tumorzellen, als In-vivo-Monitoring, als Verstärker einer Tumortherapie (Radioenhancer), als eigenständiges Therapeutikum/Medikament oder als Kombination der möglichen Eigenschaften konstruiert werden. Bei allen Möglichkeiten, die sich durch die Nanomedizin bieten, dürfen potenziell schädliche Wirkungen nicht außer Acht gelassen werden. Erst nachdem schon viele Nanopartikel entwickelt und in Umlauf gebracht worden waren, hat man begonnen, die möglichen langfristigen Folgen der massenhaften Verbreitung von Nanopartikeln wissenschaftlich zu untersuchten. Im Jahr 2004 forderten die Royal Society und die Royal Academy of Engineering, London, eine stärkere Regulierung von Nanotechnologien [17]. So gerieten beispielsweise die bereits weit verbreitete Kohlenstoffnanopartikel „carbon nanotubes“ in den Fokus, da sie sich in der Lunge anreichern und dort ähnliche Auswirkungen wie Asbest aufweisen [24]. Im gleichen Zeitraum etablierte sich schließlich auch der Begriff der Nanotoxikologie [4]. Das Verständnis von der Ausscheidung bzw. des langfristigen Verbleibs der Nanopartikel nach ihrer Aufnahme im Organismus sowie ihrer verschiedensten Nebenwirkungen auf zellulärer und subzellulärer Ebene spielt eine zunehmende Rolle in der Literatur [25]. In diesem Zusammenhang rücken aber auch immunmodulatorische Phänomene durch Nanopartikel in den Fokus. So konnte gezeigt werden, dass vermeintlich harmlose und leicht abbaubare Nanopartikel wie Calciumphosphat-Nanopartikel je nach Dosierung und Einwirkzeit zu zytotoxischen Effekten bei mesenchymalen und epithelialen Zellen der Kopf-Hals-Region und auch neuronalen Zellen führen kann (Abb. 3).

Im Gegensatz dazu wurden in einer anderen aktuellen Untersuchung humane T‑Lymphozyten mit Eisennanopartikeln, sog. „superparamagnetic iron oxide nanoparticles“ oder SPION beladen, um diese dann in einem weiteren Schritt über ein Magnetfeld in das Tumormilieu einbringen zu können. Die Viabilität und Funktionalität der T‑Zellen blieb trotz der Beladung mit den Nanopartikeln erhalten [20]. Andere experimentelle Ansätze verfolgen unter anderem die Modifikation der Programmed-Death-Ligand-1(PD-L1)-Expression oder des Toll-like-Rezeptors 9 in Tumorzellen [5, 27].

## Fazit für die Praxis

- In der Kopf-Hals-Onkologie gibt es mittlerweile eine Vielzahl von experimentellen und klinischen Studien mit Nanopartikeln.
- Diese umfassen sowohl die Diagnostik in vitro und in vivo, neue Wirkstoffe, Wirkstofftransport, Radioenhancer, Nanopartikel als eigenständige Strahlenquellen und die Kombination der verschiedenen Eigenschaften.
- Insbesondere durch diese so genannte Funktionalisierung der Nanopartikel können in Zukunft möglicherweise auch bei therapieresistenten oder kalten Tumoren weitere Fortschritte erzielt werden.
